# Environmental Risk Analysis Based on Characterization of Ground Oily Sludge

**DOI:** 10.3390/ma15249054

**Published:** 2022-12-18

**Authors:** Shifan Zhang, Jiwei Wu, Qi Nie, Xiaoxu Duan, Xianzhong Yi

**Affiliations:** 1 Cooperative Innovation Center of Unconventional Oil and Gas, Yangtze University (Ministry of Education & Hubei Province), Wuhan 430100, China; 2 School of Mechanical Engineering, Yangtze University, Jingzhou 434023, China; 3 College of Architecture & Environment, Sichuan University, Chengdu 610065, China; 4 College of Carbon Neutrality Future Technology, Sichuan University, Chengdu 610065, China

**Keywords:** energy chemical materials, oily sludge, environmental risk analysis, petroleum hydrocarbons, heavy metal, organic matter

## Abstract

Oily sludge is recognized as hazardous waste. To reduce the potential danger and harmful factors of oily sludge, it is very important to analyze its environmental risk. In this paper, the characterization of oily sludge from Shengli Oilfield in China was tested experimentally, including the composition content, particle size, microscopic morphology, heavy metal content, organic composition, inorganic composition, and thermogravimetric analysis, which were used to analyze environmental risks. The results show that the oil content of oily sludge is as high as 10.3%, which will cause serious pollution. It is calculated that China can recover 772.5 million liters of oil and reduce 553.9 million kg of carbon emissions compared with incineration in one year, if the oily sludge can be managed effectively. The content of heavy metals such as Ba, Zn, Cr, As, Ni, Se, Be, and Hg in oily sludge exceeds the standard. It will restrain the self-healing ability of soil, pollute groundwater, and endanger animals and plants. The organic matter of oily sludge is concentrated in C_11_ to C_29_. It contains a large amount of benzene series and polycyclic benzene hydrocarbons, which can lead to cancer in the human body. Inorganic substances in oily sludge are mixed with some additives, which can not only reduce the toxicity of heavy metals, but also be used as building materials. The median particle size D_50_ of oily sludge is 0.91 μm, and it spreads all over the narrow pores. Generally, it needs to be treated under high temperature conditions, which will cause secondary pollution to the environment. The research content of this paper provides a theoretical reference for the management of oily sludge.

## 1. Introduction

The global annual output of oily sludge exceeds 60 million tons, and the cumulative output exceeds 1 billion tons [1]. The annual output of oily sludge in China is as high as 6 million tons [2,3], accounting for about one-tenth of the global annual output, and the amount is still increasing year-by-year. Oily sludge cannot be directly recovered, so it may cause serious pollution and belongs to world hazardous wastes [4,5,6].

The components of oily sludge are extremely complex and mainly include emulsified oil, water, and suspended solids [3,7,8]. Its composition is related to the geological conditions, production technologies, wastewater treatment process, wastewater quality, types of chemical additives, discharge method, and management measures. Oily sludge contains many harmful and toxic substances, including chemical additives, benzene derivatives, heavy metals, and pathogens [1,9,10]. Simple landfill of oily sludge can lead to serious groundwater pollution and significant damage to natural ecosystems such as food crops, trees, and vegetation. In addition to water and solid particles containing traces of heavy metals (Cd, Cr, Cu, Ni, etc.) in oily sludge that affect the environment and human beings [11,12,13], abundant oil is also contained in oily sludge and can be further utilized [14,15]. However, due to its fine particles, flocculent shape [16,17,18], high water content, and large volume [19,20], it is not easy to realize three-phase separation of oil–water–sludge for the utilization of oily sludge.

Jahromi et al. reported that oily sludge contained 33.5% water, 14.5% light hydrocarbons, 28.0% heavy hydrocarbons, and 24.0% solids [21]. Wang et al. found that the size of particles in oily sludge was mainly concentrated around 60 μm [22]. Gao et al. indicated that dried oily sludge contained 1360.2 mg/kg Zn, 696.1 mg/kg Pb, 190.3 mg/kg Cu, 85.3 mg/kg As, 74.9 mg/kg Hg, 55.4 mg/kg Cr, 39.5 mg/kg Ni, and 2.2 mg/kg Cd [23]. Chen et al. reported that oily sludge contained 83.36% C and 11.87% H and that the content of O, N, or S did not exceed 3% [24]. Lin et al. found that oily sludge was usually composed of 40% to 52% alkanes, 28% to 31% aromatics, 8% to 10% bitumen, and 7% to 22.4% resins [25]. Gao et al. indicated that the pyrolysis process of oily sludge could be divided into three characteristic stages. The first stage is the volatilization and decomposition of light hydrocarbons at 105–350 °C, and the volatilization and decomposition of more high-boiling point organic compounds at 350–510 °C. The second stage is the secondary cracking of the previous product. The final stage (655–960 °C) is the decomposition of inorganic minerals [23,26].

**Figure 1:**
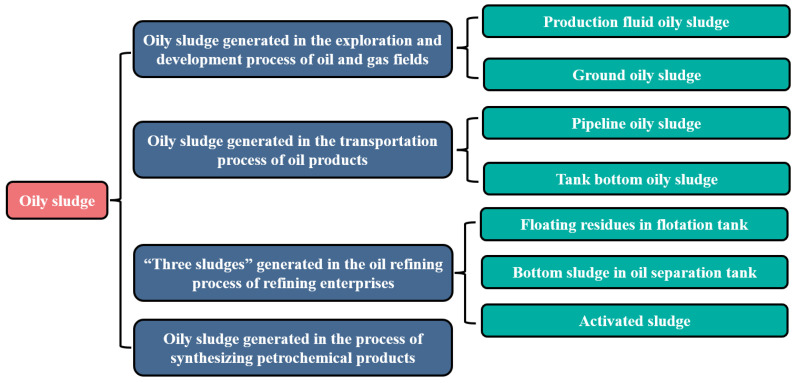
Sources and classification of oily sludge.

Oily sludge is one of the important sources of pollution generated during oil exploitation and is also one of the main factors limiting the continuous improvement in the environmental quality of oilfields. At present, oily sludge is mainly generated in the exploration, development process of oil and gas fields, the transportation process of oil products, and the oil refining process of refining enterprises [1,14], as shown in Figure 1. Based on the current production status of oily sludge, it is very important to fully understand the environmental risks brought by oily sludge. It will help oilfield managers to develop appropriate technology to deal with oily sludge and reduce its harm to the environment. This is of great significance for promoting the development of China’s “dual carbon” strategy and the sustainable development of the world.

The characterization of oily sludge samples taken from Shengli Oilfield in China was tested experimentally in this paper, including the composition content, particle size distribution, microscopic morphology, heavy metal content, organic composition, inorganic composition, and thermogravimetric analysis of oily sludge. According to the physical and chemical properties, the environmental risk of oily sludge has been discussed in many aspects, which provides support for the management of oily sludge in oilfield.

## 2. Materials and Methods

### 2.1. Materials and Experimental Methods

**Figure 2:**
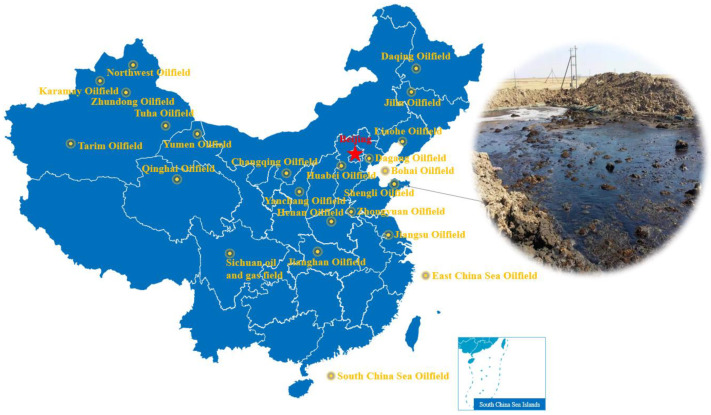
Oily sludge site in oilfield.

The oily sludge samples were from the Shengli oilfield in China; Figure 2 shows the site of oily sludge.

The density of oily sludge was determined with the weighing method. The oil content of oily sludge was determined based on Chinese oilseed meals determination of oil content (part 1: extraction method with hexane (or light petroleum); GB/T 10359-2008) [27]. The water content of oily sludge was determined based on the Chinese test method for water in petroleum products (distillation method: GB/T 260-2016) [28].

Before sending the sample to measure the particle size, the oil in the oily sludge was extracted with CCl_4_ solvent, and stirred with a magnetic stirrer, so that the extraction was uniform. The liquid phase was then filtered through qualitative filter paper. The remaining solid-phase was dried at 105 °C. An Malvern laser particle size meter (Malvern Mastersizer 3000) was used to determine the particle size of the samples (GB/T 19077-2016) [29,30]. The surface micromorphology of solid-phase particles in the sample of oily sludge was observed by a field emission scanning electron microscope (Nova NanoSEM 450) operating under an accelerating voltage of 15 kV and a detector current of 10 mA (JBT 6842-1993) [31]. The specific surface area and pore space of 0.1925 g of the sample were analyzed with a pore size analyzer (MicroActive for ASAP 2460 2.01) (SY/T 6154-2019) [32]. The density of the sample was 1 g/cm^3^ and cold free space was 51.30 cm^3^ at −195.85 °C. Before the experiment, the sample was heated at 200 °C and degassed for 6 h under vacuum conditions. A plasma emission spectrometer (Agilent 725-ES) was used to detect the contents of heavy metals in the samples (HJ 781-2016) and identify whether it was a hazardous waste (HJ 781-2016) [33,34]. The thermogravimetric analysis was performed to investigate the thermal stability and components. The thermogravimetric analyzer (TGA 8000) was heated to 800 °C at a rate of 5 °C/min under the protection of nitrogen (20 mL/min) without dwell time (GB/T 27761-2011) [35]. A gas chromatograph-mass spectrometer (GC-MS/G2577A) was used to quantify the organic components in the samples with a column (TG-5MS) with a diameter of 0.25 mm, a film thickness of 0.25 μm, and a length of 30 m. N_2_ was charged during the experiment and the temperature was heated to 300 °C (HJ 950-2018) [36]. To detect inorganic components in the samples, an XRD (X-Ray Diffraction) technique was used for the qualitative analysis (SY/T 5163-2018) [37].

### 2.2. Data Analysis Method 

With the experimental data, the density of oily sludge is calculated as:

(1) $$\rho_{{}_{1}}=\frac{M_{1}}{V_{1}}$$

where *ρ*_1_ is the density of oily sludge in g/cm^3^; $M_{1}$ is the measured mass of oily sludge in g; and $V_{1}$ is the volume of oily sludge in mL.

Oil content, water content, and solid content in oily sludge are respectively calculated as:

(2) $$\mathrm{Oil}\;\mathrm{content}=\frac{M_{1}-M_{2}}{M_{1}}\times 100\%$$

(3) $$\mathrm{Water}\;\mathrm{content}=\frac{M_{2}-M_{3}}{M_{1}}\times 100\%$$

(4) $$\mathrm{Solid}\;\mathrm{content}=\frac{M_{3}}{M_{1}}\times 100\%$$

where $M_{2}$ is the measured mass of oily sludge after extraction in g; and $M_{3}$ is the measured mass of oily sludge after drying in g.

## 3. Results and Discussion

### 3.1. Density, Oil Content, Water Content and Solid Content of Oily Sludge

The composition of oil sludge, especially the oil content, is the standard for judging hazardous waste. The environmental protection laws of various countries have clear regulations. Therefore, the oil content, water content, and solid content of the sludge were measured, and the density was also measured to support subsequent processing. The composition of oily sludge is shown in Table 1. The experimental results show that the oil content of oily sludge is 10.3%, which is 34 times the maximum oil content of 0.3% allowed by Chinese standards, 10 times that of the American standard, 5 times the Canadian standard, 21 times the French wetland standard, and 5 times the French dryland standard [38]. Based on the fact that the oil phase of oily sludge contains a lot of petroleum hydrocarbon pollutants, if it is released indiscriminately, it will cause serious pollution to the environment. After the petroleum hydrocarbons in the oil sludge seep into the soil, it will affect the permeability of the soil and reduce the quality of the soil [39]. Petroleum hydrocarbons can also hinder the respiration and absorption of plant roots, causing root rot, thereby affecting the root growth of crops [40]. After petroleum pollutants immerse into the groundwater system, they will directly affect the water resources and endanger the safety of human life [41]. The volatilization of petroleum components in oily sludge will cause the concentration of total hydrocarbons in the ambient air in the surrounding area to exceed the standard, seriously polluting the air [42].

Calculated based on the annual output of China’s oily sludge of 6 million tons and the oil content of oily sludge is 10.3%. The amount of oil in the wasted oily sludge would be as high as 618,000 tons in one year. The density of the recovered oil is calculated as 0.8 g/cm^3^; if oil could be fully recovered, China would recover 772.5 million liters. Approximately 70% of recovered oil is in the boiling point range of diesel oil and can be used as diesel fuel, which has great energy potential [43,44]. At the same time, according to the information provided by the BP China carbon emission calculator, saving 1 L of diesel oil is equivalent to reducing 2.63 kg of carbon dioxide emissions, which is equivalent to reducing 0.717 kg of carbon emissions. Compared with direct incineration, the recycling of the oil in oily sludge can ultimately reduce 553.9 million kg of carbon emissions if all recovered oil is calculated as diesel oil, which can reduce greenhouse gas emissions and slow down global warming. This will contribute to the early achievement of China’s “dual carbon” goals and contribute to the promotion of global sustainable development.

### 3.2. Particle Size Distribution of Oily Sludge

**Figure 3:**
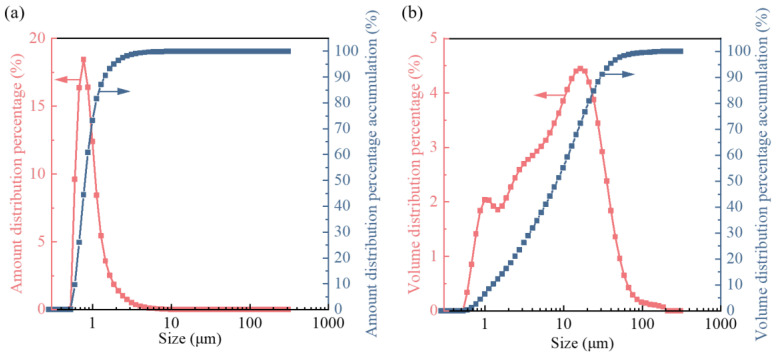
Particle size distribution of oily sludge: (**a**) amount distribution; (**b**) volume distribution.

The particle size of oily sludge particles directly affects the adhesion of pollutants, the degree of diffusion, and the difficulty of subsequent treatment. Therefore, the particle size distribution analysis of the oil sludge sample was carried out. It can be seen from Figure 3 that the particle size of oily sludge ranges from 0.1 to 309.53 μm, the volume average particle size is 14.79 μm, and the surface area average particle size is 4.127 μm. In the number density distribution shown in Figure 3a, D_10_ is 0.68 μm, median particle size D_50_ is 0.91 μm, and D_100_ is 22.78 μm; in the bulk density distribution Figure 3b, D_10_ is 1.41 μm, median particle size D_50_ is 9.41 μm and D_100_ is 209.62 μm. Due to the small particle size, the oil adheres to the surface of the tiny particles, the bonding specific surface area between the oil and the solid particles is large, the bonding force is strong, and it is relatively difficult to separate oil from solid particles [45]. It can be seen that under non-high temperature conditions, the oil phase in oily sludge is difficult to be completely removed. However, under high temperature conditions, to completely remove the oil phase in the oily sludge, the energy consumption of the equipment will increase accordingly, and the secondary pollution generated in the high-temperature environment will cause serious pollution to the environment.

### 3.3. Micromorphological Characteristics, Specific Surface Area and Porosity of Oily Sludge

**Figure 4:**
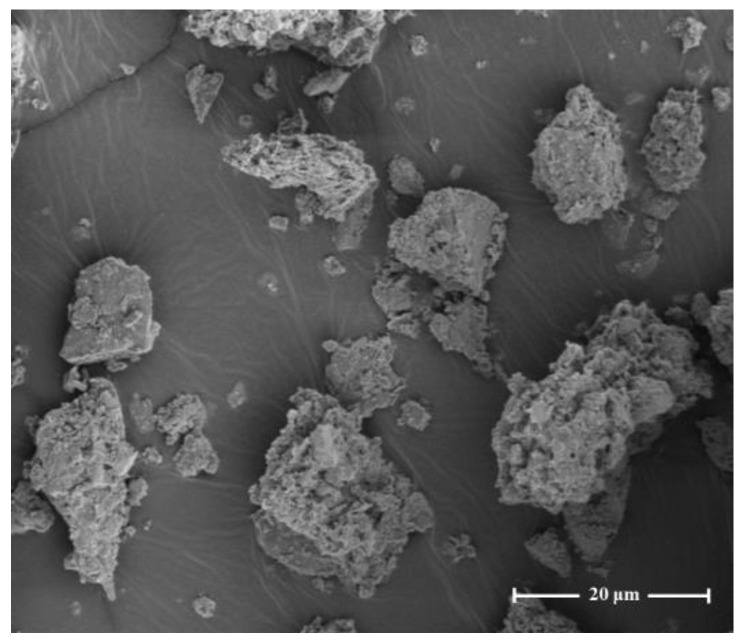
SEM microscopy of oily sludge.

Oily sludge is a porous medium. Surface structure and pore distribution will directly affect the distribution and pollution of pollutants in oily sludge. Therefore, it is necessary to understand the surface morphology and pore distribution of oily sludge. Figure 4 shows the micromorphology of oily sludge. Solid-phase particles are irregular in shape and rough in the surface. Pores and cracks can be observed in the surface and internal tunnel [46]. Zhao et al. [47] studied the morphology changes of oily sludge during solvent extraction by scanning electron microscopy (SEM). It was found that with the extension of the extraction time, the oil gradually separated from the surface, and a large number of pore structures gradually emerged, which further verified that the oily sludge is a porous structure.

**Figure 5:**
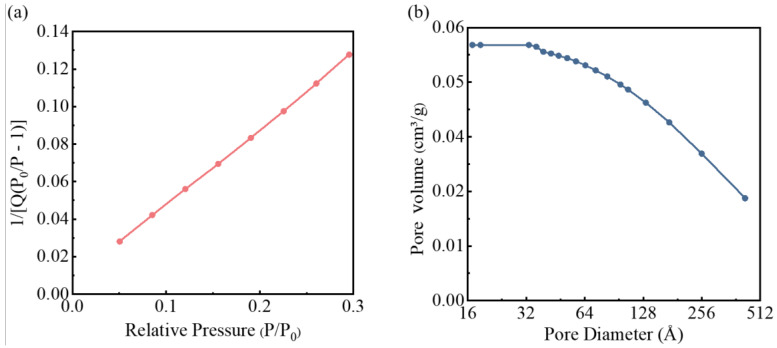
Pore diameter distribution and specific surface area of oily sludge: (**a**) BET surface area plot; (**b**) BJH desorption cumulative pore volume.

The porous structure characteristics of solid-phase particles in oily sludge were analyzed by the Brunner-Emmett-Teller (BET) method [48]. According to the BJH method, the BET surface area is 10.59 m^2^/g, as shown in Figure 5a; the pore volume is 0.056 cm^3^/g, and the average pore size of the pores is 169.29 Å, as shown in Figure 5b. The analysis results show that the pores of oily sludge particles are narrow, and part of the oil phase exists in the pores, which is called pore oil. Pore oil adheres to the solid surface and is in equilibrium under capillary forces. To achieve the removal of pore oil, it is necessary to overcome the capillary force. However, deoiling oil in such small pores is extremely challenging. As a result, it is difficult to manage oilfield pollutants, which will further increase the risk of environmental pollution.

### 3.4. Contents of Heavy Metals in Oily Sludge

**Figure 6:**
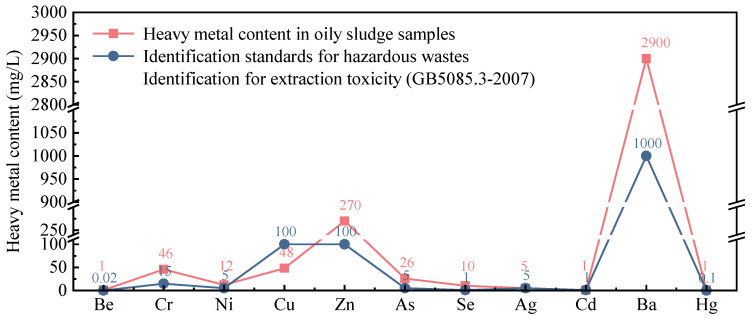
Heavy metal content of oily sludge.

Heavy metals have a certain toxicity, which is an important indicator to measure the degree of environmental pollution. Therefore, the heavy metal content of oily sludge was determined to detect whether the content exceeds the standard. The content of heavy metals in the oily sludge leaching solution is shown in Figure 6. The concentration sequence of heavy metals in oily sludge is Ba (2900 mg/L) > Zn (270 mg/L) > Cu (48 mg/L) > Cr (46 mg/L) > As (26 mg/L) > Ni (12 mg/L) > Se (10 mg/L) > Ag (5 mg/L) > (Be, Cd, Hg) [49]. According to the Chinese national standard (GB5085.3-2007) [50], the leaching upper limits corresponding to heavy metal elements Ba, Zn, Cr, As, Ni, Se, Be, and Hg are 1000 mg/L, 100 mg/L, 15 mg/L, 5 mg/L, 5 mg/L, 1 mg/L, 0.02 mg/L, and 0.1 mg/L, respectively. In contrast, the heavy metal content in oily sludge is high, and the concentrations of Ba, Zn, Cr, As, Ni, Se, Be, and Hg all exceed the limit, which will cause serious pollution to the ecological environment.

The heavy metal substances in oily sludge flow into the cultivated land, which will cause great harm to the cultivated land. Heavy metal pollution of cultivated land seriously reduces the content of beneficial bacteria in the soil, greatly reducing the ability of self-repair and self-regulation of cultivated land. Severe heavy metal pollution in cultivated land will reduce grain production, affect the quality of agricultural products, and endanger the healthy growth of plants, animals, and humans. In addition, heavy metals flow into the surface water with the water flow and infiltrate into the groundwater, which will cause the COD, BOD, and other indicators of the groundwater to seriously exceed the standard.

### 3.5. Analysis of Organic Compounds in Oily Sludge

**Figure 7:**
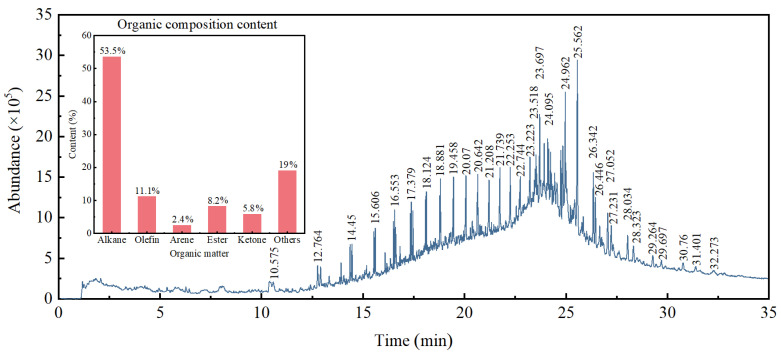
The spectrum of organic matters in oily sludge.

Organic pollutants are the main components of oily sludge and are extremely harmful. Therefore, it is necessary to measure the organic composition of oily sludge to support the subsequent treatment. The specific composition of oily sludge is shown in Appendix A. It can be seen from the map analysis in Figure 7 that the abundance distribution is 130 peaks. The organic matter in the oily sludge is concentrated in C_11_–C_29_ (carbon atoms, *n* = 1, 2, 3…), of which alkanes account for 53.5%, olefins account for 11.1%, aromatic hydrocarbons account for 2.4%, esters account for 8.2%, ketones account for 5.8%, and other ingredients account for 19%. Oily sludge contains a large amount of toxic and harmful substances such as benzene series and polycyclic benzene hydrocarbons (Polycyclic Aromatic Hydrocarbons, PAHs). Benzene series are highly toxic and carcinogenic. It can enter the human body through the respiratory tract, digestive tract, and skin, and has a greater correlation with the high incidence of leukemia. Toluene and xylene are highly toxic to the human central nervous system and blood system, and other benzene series such as ethylbenzene are also harmful to the human body. As an organic component of oily sludge, PAHs are widely distributed in the oilfield environment, including air, water, or soil, with strong stability and high bioaccumulation rate. Additionally, they are potentially carcinogenic to humans. Mutation and endocrine disruption pose a threat to human health and the ecological environment, which has aroused widespread concern around the world. In 1979, USEPA listed 16 kinds of PAHs without branches as priority pollutants in the environment, and China also included PAHs in the blacklist of environmental pollution [51,52].

### 3.6. Analysis of Inorganic Compounds in Oily Sludge

**Figure 8:**
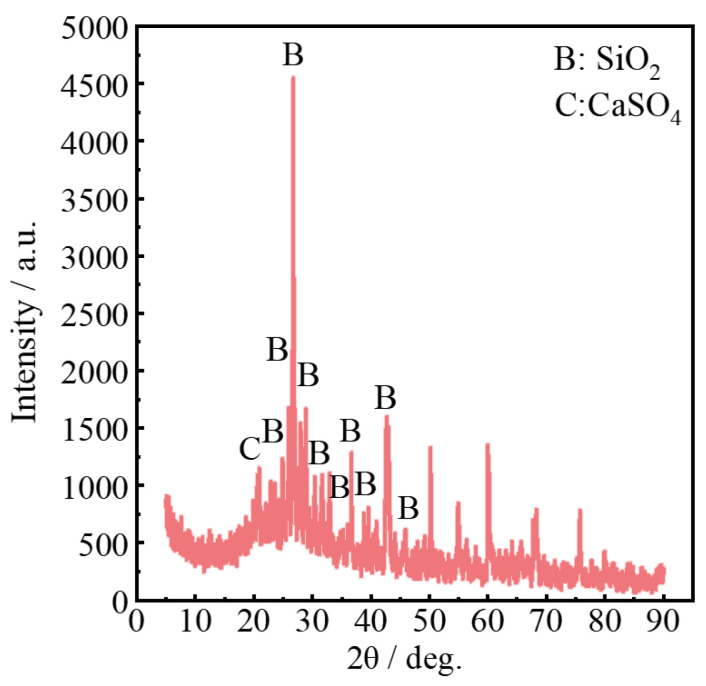
Spectrum of inorganic compounds in oily sludge.

The inorganic matter of oily sludge needs to be utilized as an environmental resource. Therefore, it is necessary to detect the inorganic composition of oily sludge to provide a basis for its resource recycling. Inorganic compounds in oily sludge included SiO_2_ and a small quantity of CaSO_4_, as shown in Figure 8. These inorganic components could be applied to build materials, including paving substrates, cement clinker, non-fired ceramic granules, non-fired bricks, light aggregates, and sintered ceramics. The key to the utilization of building materials is to adopt measures to local conditions. The utilization method should be chosen according to the local geographical environment and actual needs. It is necessary to ensure the performance of the material and meet the requirements of environmental protection. Taha et al. used various additives to solidify the oily sludge at the bottom of the tank and evaluated the strength and toxicity leaching after solidification. The cured material could be used in roads, dams, and landfills in building construction [53]. Xiao et al. used phosphogypsum-based cementitious materials to recycle oily sludge as roadbed materials, which greatly reduced the content of heavy metals and enhanced environmental protection [54].

### 3.7. Thermogravimetric Analysis of Oily Sludge

**Figure 9:**
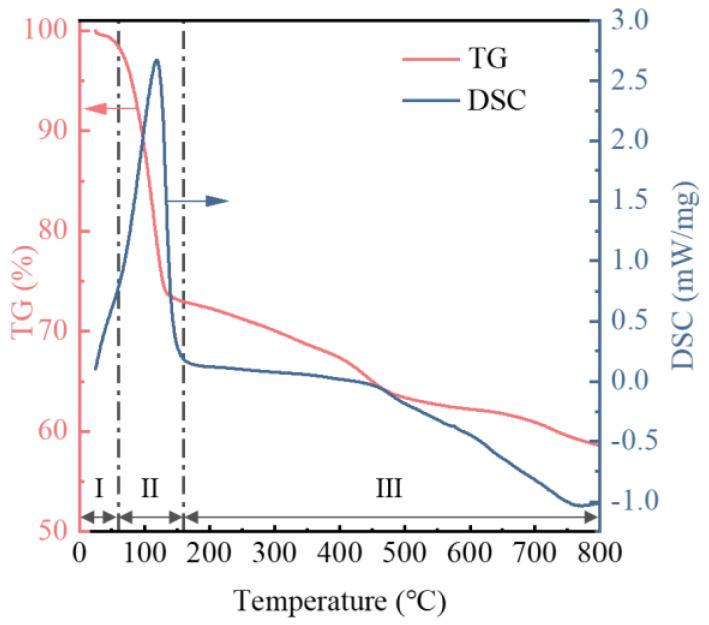
Thermogravimetric curves of oily sludge.

Oily sludge is composed of oil, water, and solids. Understanding the thermal stability of oily sludge under high-temperature conditions can help reduce its secondary pollution. Therefore, thermogravimetric analysis was performed on the oily sludge. The thermogravimetric TG curve and DSC curve of oily sludge are shown in Figure 9. The pyrolysis process of oily sludge can be divided into three stages: below 60 °C (I), 60–160 °C (II), and 160–800 °C (III). In the first stage (I), the slight weight loss is mainly caused by the evaporation of water attached to the surface of oily sludge. In the second stage (II), it is mainly the volatilization of water in oily sludge and a small amount of light oil. At this stage, the DSC curve has a strong endothermic peak, which further indicates that a large amount of volatilization of the adsorbed water in the oily sludge leads to a large range of weight loss. In the third stage (III), it is mainly caused by the volatile cracking of light oil and heavy oil, and the decomposition of organic salts in oily sludge. Overall, the total weight loss of oily sludge was 41.4%, and the corresponding residual weight was 58.6%, which is almost consistent with the composition content results in Table 1. Therefore, when the oil phase in the oily sludge is separated by the thermal separation method, the temperature requirement is relatively high. Taking the thermal desorption method as an example, the general temperature is controlled between 350–480 °C [55]. As a result, the secondary pollutants and energy consumption of equipment operation increase, and the risk of pollution to the environment will also increase accordingly. At the same time, it is necessary to collect and treat secondary pollutants, which can reduce environmental pollution.

## 4. Conclusions

In this paper, the characterization of oily sludge was tested experimentally to analyze its risk to the environment. The following conclusions were obtained:

The oil content of oily sludge is as high as 10.3%, so it is deduced that the amount of oil in the oily sludge produced in China in one year is as high as 618,000 tons. A large amount of petroleum hydrocarbon pollutants will seriously affect the properties of soil, endanger the growth and development of plants, and pollute water bodies and air. If the oil is recycled, it can reduce carbon emissions and slow down the greenhouse effect.

In the number density distribution, the median diameter D_50_ of oily sludge particles is 0.91 μm. Due to the small particle size, the oil adheres to the surface of the tiny particles, the bonding specific surface area between the oil and the solid particles is large, the bonding force is strong, and it is relatively difficult to separate oil from solid particles. If the oil phase is separated under high-temperature conditions, it will increase the energy consumption of the equipment and cause secondary pollution to the environment. At the same time, Oily sludge has a porous structure with narrow pores, and the pore oil is difficult to remove under the action of capillary forces. It is difficult to control oilfield pollutants, and the risk of environmental pollution has further increased.

The concentration sequence of heavy metals in oily sludge is Ba (2900 mg/L) > Zn (270 mg/L) > Cu (48 mg/L) > Cr (46 mg/L) > As (26 mg/L) > Ni (12 mg/L) > Se (10 mg/L) > Ag (5 mg/L) > (Be, Cd, Hg). The concentrations of Ba, Zn, Cr, As, Ni, Se, Be, and Hg all exceed the limit, which will cause serious pollution to soil and groundwater.

The organic compounds in the oily sludge are concentrated in C_11_–C_29_. Benzene series and PAHs are widely distributed in the air, water, and soil of oil fields, and are carcinogenic to humans. Inorganic compounds in oily sludge included SiO_2_ and a small quantity of CaSO_4_, which can be used in construction materials after adding additives. It not only reduces its toxicity and realizes waste utilization, but also reduces the risk of environmental pollution.

Based on the research in this paper, an evaluation system for oily sludge treatment technology can be constructed in the future. For the oily sludge in a specific area, the analytic hierarchy process (AHP), fuzzy comprehensive evaluation (FCE), and life cycle assessment (LCA) methods are used to select the appropriate treatment technology, including high-temperature incineration, chemical extraction, thermal analysis, and bioremediation. It provides a theoretical reference for oilfield personnel to manage oily sludge scientifically.

## Figures and Tables

**Table 1:** Composition of oily sludge.

Indexes	Density (g/cm^3^)	Oil Content (%)	Water Content (%)	Solid Content (%)
Values	1.75	10.3	29.9	59.8

## Data Availability

Data presented in this article are available on request from the corresponding author.

## Appendix A

**Table A1:** Analysis results of organic components of oily sludge.

Retention Time (min)	Products	Molecular Formulas	Relative Peak Areas (%)	Categories	CAS
1.179	Sulfur dioxide	O_2_S	0.23	Other	007446-09-5
1.248	Carbon disulfide	CS_2_	0.09	Other	000075-15-0
1.277	Thiourea	CH_4_N_2_S	0.09	Other	000062-56-6
1.416	3-Chlorohexane	C_6_H_13_Cl	0.15	Other	002346-81-8
1.514	2-cyclopropyl-Pentane	C_8_H_16_	0.06	Alkanes	005458-16-2
1.583	1-Heptene	C_7_H_14_	0.06	Olefin	000592-76-7
10.39	1-Tridecene	C_13_H_26_	0.17	Olefin	002437-56-1
10.448	1-Tetradecene	C_14_H_28_	0.16	Olefin	001120-36-1
10.581	Tridecane	C_13_H_28_	0.27	Alkanes	000629-50-5
12.764	2-Tetradecene, (E)-	C_14_H_28_	0.32	Olefin	035953-53-8
12.908	Tetradecane	C_14_H_30_	0.29	Alkanes	000629-59-4
13.33	2,6-dimethyl-Naphthalene	C_12_H_12_	0.11	Aromatics	000581-42-0
13.844	2-hexyl-1-Decanol	C_16_H_34_O	0.08	Other	2425-77-6
13.919	Decane, 2-methyl-	C_11_H_24_	0.18	Alkanes	006975-98-0
14.052	2-Buten-1-one, 1-(2,6,6-trimethyl-3-cyclohexen-1-yl)-	C_13_H_20_O	0.09	Ester	041436-42-4
14.358	1-Pentadecene	C_15_H_30_	0.28	Olefin	013360-61-7
14.456	Pentadecane	C_15_H_32_	0.3	Alkanes	000629-62-9
15.092	Naphthalene, 1,6,7-trimethyl-	C_13_H_14_	0.05	Aromatics	002245-38-7
15.178	Magnesium, bis(acetylacetonate)	C_10_H_14_MgO_4_.2(H_2_O)	0.1	Other	068488-07-3
15.536	2-Tetradecene, (E)-	C_14_H_28_	0.33	Olefin	035953-53-8
15.606	Hexadecane	C_16_H_34_	0.3	Alkanes	000544-76-3
16.091	Pentadecane, 2,6,10-trimethyl-	C_18_H_38_	0.22	Alkanes	003892-00-0
16.172	3-Methyl-4-(methoxycarbonyl)hexa-2,4-dienoic acid	C_9_H_12_O_4_	0.12	Ester	1000104-10-8
16.224	4-n-Hexylthiane	C_11_H_22_S	0.13	Other	070928-52-8
16.345	4-n-Hexylthiane	C_11_H_22_S	0.22	Other	070928-52-8
16.403	4-n-Hexylthiane	C_11_H_22_S	0.11	Other	070928-52-8
16.495	1-Heptadecene	C_17_H_34_	0.11	Olefin	006765-39-5
16.558	Heptadecane	C_17_H_36_	0.43	Alkanes	000629-78-7
16.605	Pentadecane, 2,6,10,14-tetramethyl	C_19_H_40_	0.39	Alkanes	001921-70-6
16.639	.beta.-iso-Methyl ionone	C_14_H_22_O	0.18	Ketone	1000285-40-2
16.882	4-n-Hexylthiane	C_11_H_22_S	0.15	Other	070928-52-8
17.09	Silane, dichlorocyclohexylmethyl-	C_7_H_14_Cl_2_Si	0.28	Other	005578-42-7
17.286	Cyclopentane, 1-butyl-2-pentyl-	C_14_H_28_	0.29	Alkanes	061142-52-7
17.332	1-Octadecene	C_18_H_36_	0.43	Olefin	000112-88-9
17.384	Octadecane	C_18_H_38_	0.61	Alkanes	000593-45-3
17.459	Hexadecane, 2,6,10,14-tetramethyl-	C_20_H_42_	0.5	Alkanes	000638-36-8
17.65	3-Eicosene, (E)-	C_20_H_40_	0.43	Olefin	074685-33-9
17.76	Cyclohexanebutanoic acid, 2,2-dimethyl-6-methylene-, methyl ester	C_14_H_24_O_2_	0.32	Ester	095452-15-6
17.852	1H-Indene, 5-butyl-6-hexyloctahydro	C_19_H_36_	0.45	Alkanes	055044-36-5
17.904	2-Butanone, 4-(2,2,6-trimethylcyclohexyl)-	C_13_H_24_O	0.19	Ketone	006138-85-8
18.002	Cyclohexene, 4-(4-ethylcyclohexyl)-1-pentyl-	C_19_H_34_	0.002	Alkanes	301643-32-3
18.083	1-Nonadecene	C_19_H_38_	0.81	Olefin	018435-45-5
18.129	Heptadecane	C_17_H_36_	0.56	Alkanes	000629-78-7
18.176	8-Hexadecyne	C_16_H_30_	0.3	Other	019781-86-3
18.228	1H-Indene, 2-butyl-5-hexyloctahydro-	C_19_H_36_	0.23	Alkanes	055044-33-2
18.297	1-Octadecene	C_18_H_36_	0.41	Olefin	000112-88-9
18.337	11,13-Dimethyl-12-tetradecen-1-olacetate	C_18_H_34_O_2_	0.36	Ester	1000130-81-0
18.47	Phenanthrene, 4-methyl-	C_15_H_12_	0.39	Aromatics	000832-64-4
18.511	Phenanthrene, 1-methyl-	C_15_H_12_	0.34	Aromatics	000832-69-9
18.563	2-Dodecen-1-yl(-)succinic anhydrid	C_16_H_26_O_3_	0.35	Other	019780-11-1
18.643	Cyclohexane, 1-(1,5-dimethylhexyl)-4-(4-methylpentyl)-	C_20_H_40_	0.27	Other	056009-20-2
18.707	2-Eicosanol, (.+/−.)-	C_20_H_42_O	0.42	Other	4340-76-5
18.776	1-Nonadecene	C_19_H_38_	0.76	Olefin	018435-45-5
18.817	Eicosane	C_20_H_42_	0.67	Alkanes	000112-95-8
18.949	Cyclotetradecane, 1,7,11-trimethyl	C_20_H_40_	0.55	Alkanes	001786-12-5
19.082	11,13-Dimethyl-12-tetradecen-1-olacetate	C_18_H_34_O_2_	0.38	Ester	1000130-81-0
19.209	11,13-Dimethyl-12-tetradecen-1-olacetate	C_18_H_34_O_2_	0.39	Ester	1000130-81-0
19.25	Phenanthrene, 3,6-dimethyl-	C_16_H_14_	0.35	Aromatics	001576-67-6
19.325	Oxirane, hexadecyl-	C_18_H_36_O	0.81	Other	007390-81-0
19.429	1-Nonadecene	C_19_H_38_	0.8	Olefin	018435-45-5
19.463	Heneicosane	C_21_H_44_	0.81	Alkanes	000629-94-7
19.596	Z-8-Methyl-9-tetradecen-1-ol acetate	C_17_H_32_O_2_	0.5	Ester	1000130-82-4
19.671	Oxirane, tridecyl-	C_15_H_30_O	0.47	Other	018633-25-5
19.706	Cyclotetradecane, 1,7,11-trimethyl-4-(1-methylethyl)-	C_20_H_40_	0.51	Alkanes	001786-12-5
19.781	Cyclohexene, 1-pentyl-4-(4-propylcyclohexyl)-	C_20_H_36_	0.4	Olefin	108067-20-5
19.833	1H-Indene, 5-butyl-6-hexyloctahydro-	C_19_H_36_	0.46	Alkanes	055044-36-5
19.954	E-8-Methyl-9-tetradecen-1-ol acetate	C_17_H_32_O_2_	0.63	Ester	1000130-81-4
20.041	1-Nonadecene	C_19_H_38_	0.71	Olefin	018435-45-5
20.07	Docosane	C_22_H_46_	0.87	Alkanes	000629-97-0
20.289	Hexadecane, 2-methyl-	C_17_H_36_	0.6	Alkanes	001560-92-5
20.382	Pregnane	C_21_H_36_	0.68	Alkanes	000481-26-5
20.526	9-Cedranone	C_15_H_24_O	0.55	Ketone	1000156-23-2
20.624	9-Tricosene, (Z)-	C_23_H_46_	0.79	Olefin	027519-02-4
20.653	Tricosane	C_23_H_48_	0.86	Alkanes	000638-67-5
20.803	Cyclohexene, 4-(4-ethylcyclohexyl)-1-pentyl-	C_19_H_34_	0.37	Alkanes	301643-32-3
20.85	10,13-Octadecadienoic acid, methylester	C_19_H_34_O_2_	0.46	Other	056554-62-2
21.034	9,12-Octadecadienoic acid (Z,Z)-,methyl ester	C_19_H_34_O_2_	0.77	Other	000112-63-0
21.184	1-Hexacosene	C_26_H_52_	0.82	Olefin	018835-33-1
21.208	Tetracosane	C_24_H_50_	0.81	Alkanes	000646-31-1
21.329	Tricosane	C_23_H_48_	0.77	Alkanes	000638-67-5
21.444	1-Docosanol, acetate	C_24_H_48_O_2_	0.54	Ester	000822-26-4
21.537	1-Bromo-11-iodoundecane	C_11_H_22_BrI	0.54	Other	139123-69-6
21.635	Cyclohexene, 4-(4-ethylcyclohexyl)-1-pentyl-	C_19_H_34_	0.53	Alkanes	301643-32-3
21.739	Docosane	C_22_H_46_	2.01	Alkanes	000629-97-0
22.033	Octadecane, 2,6,10,14-tetramethyl-	C_22_H_46_	0.66	Alkanes	054964-82-8
22.253	Hexacosane	C_26_H_54_	1.59	Alkanes	000630-01-3
22.449	1,1′:3′,1″-Tercyclopentane, 2′-dodecyl-	C_27_H_50_	1.1	Alkanes	055282-68-3
22.547	1-Bromo-11-iodoundecane	C_11_H_22_BrI	1.32	Other	139123-69-6
22.686	11,12-Dibromo-tetradecan-1-ol acetate	C_16_H_30_Br_2_O_2_	1	Ester	1000130-78-5
22.75	Hexacosane	C_26_H_54_	1.47	Other	000630-01-3
22.83	11,12-Dibromo-tetradecan-1-ol acetate	C_16_H_30_Br_2_O_2_	0.67	Other	1000130-78-5
22.865	11,12-Dibromo-tetradecan-1-ol acetate	C_16_H_30_Br_2_O_2_	0.46	Ester	1000130-78-5
22.888	Octadecanoic acid, 2-oxo-, methyl ester	C_19_H_36_O_3_	0.55	Ester	002380-18-9
22.952	2-Dodecen-1-yl(-)succinic anhydrid	C_16_H_26_O_3_	0.88	Other	019780-11-1
23.085	1H-Indene, 5-butyl-6-hexyloctahydro-	C_19_H_36_	0.5	Alkanes	055044-36-5
23.223	Hexacosane	C_26_H_54_	2.24	Alkanes	000630-01-3
23.316	1,1′:3′,1″-Tercyclopentane, 2′-dodecyl-	C_27_H_50_	0.72	Alkanes	055282-68-3
23.373	1H-Indene, 5-butyl-6-hexyloctahydro-	C_19_H_36_	1.11	Alkanes	055044-36-5
23.483	Cholestane	C_27_H_48_	0.82	Alkanes	000481-21-0
23.523	Cholestane	C_27_H_48_	1.12	Alkanes	000481-21-0
23.564	1H-Indene, 5-butyl-6-hexyloctahydro-	C_19_H_36_	0.82	Alkanes	055044-36-5
23.604	1H-Indene, 5-butyl-6-hexyloctahydro-	C_19_H_36_	1	Alkanes	055044-36-5
23.703	Cholestane	C_27_H_48_	2.21	Alkanes	000481-21-0
23.755	3,7-Dimethyl-6-nonen-1-ol acetate	C_13_H_24_O_2_	1.2	Ester	1000131-34-9
23.858	(-)-Neoclovene-(I), dihydro-	C_15_H_26_	1.34	Olefin	1000152-82-1
23.893	1H-Indene, 5-butyl-6-hexyloctahydro-	C_19_H_36_	0.66	Alkanes	055044-36-5
24.055	Cholestane	C_27_H_48_	1.09	Alkanes	000481-21-0
24.101	Cholestan-3-one	C_27_H_46_O	1.33	Alkanes	015600-08-5
24.153	Silane, dimethyldi(2-propylphenoxy)-	C_20_H_28_O_2_Si	1.17	Other	1000347-41-8
24.292	Cholestane	C_27_H_48_	1.04	Alkanes	000481-21-0
24.355	1H-Indene, 5-butyl-6-hexyloctahydro-	C_19_H_36_	0.97	Alkanes	055044-36-5
24.436	Stigmastane	C_29_H_52_	2.14	Alkanes	000601-58-1
24.552	Stigmastane	C_29_H_52_	2.09	Alkanes	000601-58-1
24.846	Stigmastane	C_29_H_52_	1.99	Alkanes	000601-58-1
25.042	1H-Indene, 5-butyl-6-hexyloctahydro-	C_19_H_36_	2.02	Alkanes	055044-36-5
25.216	1H-Indene, 5-butyl-6-hexyloctahydro-	C_19_H_36_	0.8	Alkanes	055044-36-5
25.343	2,6-Diphenylpyridine	C_17_H_13_N	0.54	Other	003558-69-8
25.429	Benzophenone, 2-methylamino-5-chloro-	C_14_H_12_ClNO	1.52	Other	074966-83-9
25.562	1-Penten-3-one, 1-(2,6,6-trimethyl-1-cyclohexen-1-yl)-	C_14_H_22_O	3.22	Ketone	000127-43-5
25.782	Sophocarpine, N-oxide	C_15_H_22_N_2_O_2_	0.76	Other	26904-64-3
26.018	Lanosterol	C_30_H_50_O	0.45	Other	000079-63-0
26.758	Bromacil	C_9_H_13_BrN_2_O_2_	0.39	Other	000314-40-9
27.058	Anthracene, 9-butyl-	C_18_H_18_	0.75	Aromatics	001498-69-7
27.329	Tricosane	C_23_H_48_	0.25	Alkanes	000638-67-5
28.04	1-Penten-3-one, 1-(2,6,6-trimethyl-1-cyclohexen-1-yl)-	C_14_H_22_O	0.36	Ketone	000127-43-5
28.323	1-Penten-3-one, 1-(2,6,6-trimethyl-1-cyclohexen-1-yl)-	C_14_H_22_O	0.25	Ketone	000127-43-5
29.27	1,2-Bis(trimethylsilyl)benzene	C_12_H_22_Si_2_	0.2	Other	017151-09-6
29.703	Benzo[h]quinoline, 2,4-dimethyl-	C_15_H_13_N	0.18	Other	000605-67-4
30.766	2-(Acetoxymethyl)-3-(methoxycarbonyl)biphenylene	C_17_H_14_O_4_	0.16	Ester	093103-70-9

## References

[1] Wang Z., Gong Z., Wang Z., Li X., Chu Z. (2021). Application and development of pyrolysis technology in petroleum oily sludge treatment. Environ. Eng. Res..

[2] Qin Y., Zhang K., Wu X., Ling Q., Hu J., Li X., Liu H. (2021). Effect of Oily Sludge Treatment with Molten Blast Furnace Slag on the Mineral Phase Reconstruction of Water-Quenched Slag Properties. Materials.

[3] Hui K., Tang J., Lu H., Xi B., Qu C., Li J. (2020). Status and prospect of oil recovery from oily sludge: A review. Arab. J. Chem..

[4] Liu H., Li S., Guo G., Gong L., Zhang L., Qie Y., Hu H., Yao H. (2021). Ash formation and the inherent heavy metal partitioning behavior in a 100 t/d hazardous waste incineration plant. Sci. Total Environ..

[5] Jiang G., Li J., Yu J., Jiang H., Li H., Xu B., Zhao L., Wang H. (2021). Research on the influencing factors and mechanism of single-phase microemulsion cleaning of shale gas oil-based cuttings. Environ. Technol..

[6] Wu X., Yue B., Su Y., Wang Q., Huang Q., Wang Q., Cai H. (2017). Pollution characteristics of polycyclic aromatic hydrocarbons in common used mineral oils and their transformation during oil regeneration. J. Environ. Sci..

[7] Zhao C., Zhou J., Yan Y., Yang L., Xing G., Li H., Wu P., Wang M., Zheng H. (2021). Application of coagulation/flocculation in oily wastewater treatment: A review. Sci. Total Environ..

[8] Liu J., Zhang Y.-X., Peng K.-M., Zhao X., Xiong Y., Huang X.-F. (2021). A review of the interfacial stability mechanism of aging oily sludge: Heavy components, inorganic particles, and their synergism. J. Hazard. Mater..

[9] Wang J., Liu T.-L., Huang Q.-X., Ma Z.-Y., Chi Y., Yan J.-H. (2017). Production and characterization of high quality activated carbon from oily sludge. Fuel Process. Technol..

[10] Jin X., Teng D., Fang J., Liu Y., Jiang Z., Song Y., Zhang T., Siyal A.A., Dai J., Fu J. (2021). Petroleum oil and products recovery from oily sludge: Characterization and analysis of pyrolysis products. Environ. Res..

[11] Jasmine J., Mukherji S. (2015). Characterization of oily sludge from a refinery and biodegradability assessment using various hydrocarbon degrading strains and reconstituted consortia. J. Environ. Manag..

[12] Li J., Lin F., Xiang L., Zheng F., Che L., Tian W., Guo X., Yan B., Song Y., Chen G. (2021). Hazardous elements flow during pyrolysis of oily sludge. J. Hazard. Mater..

[13] Wan G., Bei L., Yu J., Xu L., Sun L. (2022). Products distribution and hazardous elements migration during pyrolysis of oily sludge from the oil refining process. Chemosphere.

[14] Li J., Lin F., Li K., Zheng F., Yan B., Che L., Tian W., Chen G., Yoshikawa K. (2021). A critical review on energy recovery and non-hazardous disposal of oily sludge from petroleum industry by pyrolysis. J. Hazard. Mater..

[15] Hu G., Feng H., He P., Li J., Hewage K., Sadiq R. (2020). Comparative life-cycle assessment of traditional and emerging oily sludge treatment approaches. J. Clean. Prod..

[16] Gao N., Duan Y., Li Z., Quan C., Yoshikawa K. (2021). Hydrothermal treatment combined with in-situ mechanical compression for floated oily sludge dewatering. J. Hazard. Mater..

[17] Gao N., Li J., Quan C., Wang X., Yang Y. (2020). Oily sludge catalytic pyrolysis combined with fine particle removal using a Ni-ceramic membrane. Fuel.

[18] Rahbari-Sisakht M., Pouranfard A., Darvishi P., Ismail A.F. (2017). Biosurfactant production for enhancing the treatment of produced water and bioremediation of oily sludge under the conditions of Gachsaran oil field. J. Chem. Technol. Biotechnol..

[19] Deng S., Wang X., Tan H., Mikulčić H., Yang F., Li Z., Duić N. (2016). Thermogravimetric study on the co-combustion characteristics of oily sludge with plant biomass. Thermochim. Acta.

[20] Johnson O.A., Affam A.C. (2019). Petroleum sludge treatment and disposal: A review. Environ. Eng. Res..

[21] Fellah Jahromi A., Elektorowicz M. (2018). Electrokinetically assisted oil-water phase separation in oily sludge with implementing novel controller system. J. Hazard. Mater..

[22] Wang J., Han X., Huang Q., Ma Z., Chi Y., Yan J. (2018). Characterization and migration of oil and solids in oily sludge during centrifugation. Environ. Technol..

[23] Gao N., Li J., Quan C., Tan H. (2020). Product property and environmental risk assessment of heavy metals during pyrolysis of oily sludge with fly ash additive. Fuel.

[24] Chen L., Zhang X., Sun L., Xie X., Yang S., Mei N. (2019). Study on the fast pyrolysis of oil sludge by PY-GC/MS. Pet. Sci. Technol..

[25] Lin B., Huang Q., Chi Y. (2018). Co-pyrolysis of oily sludge and rice husk for improving pyrolysis oil quality. Fuel Process. Technol..

[26] Vamvuka D., Salpigidou N., Kastanaki E., Sfakiotakis S. (2009). Possibility of using paper sludge in co-firing applications. Fuel.

[27] (2008). Determination of Oil Content in Oil Cake Part 1: Hexane (or Petroleum Ether) Extraction Method.

[28] (2016). Distillation Method for Determination of Water Content in Petroleum Products.

[29] Kubínová R., Neumann M., Kavka P. (2021). Aggregate and particle size distribution of the soil sediment eroded on steep artificial slopes. Appl. Sci..

[30] Zhang L., Zhang A., Li K., Wang Q., Han Y., Yao B., Gao X., Feng L. (2020). Research on the pretreatment and mechanical performance of undisturbed phosphogypsum. Case Stud. Constr. Mater..

[31] Generali L., Malovo A., Bolelli G., Borghi A., La Rosa G.R.M., Puddu P., Lusvarghi L., Rota A., Consolo U., Pedullà E. (2020). Mechanical properties and metallurgical features of new green NiTi reciprocating instruments. Materials.

[32] Miao Y., Luo H., Pudukudy M., Zhi Y., Zhao W., Shan S., Jia Q., Ni Y. (2020). CO_2_ capture performance and characterization of cellulose aerogels synthesized from old corrugated containers. Carbohydr. Polym..

[33] Xin L., Jihong Q., Hui S., Zhiwei G., Wenqing C., Zhi L. (2021). Leaching of heavy metals and their impacting factors from a spent catalyst in the refinery industry. Environ. Chem..

[34] Deng L., Yao B., Lu W., Zhang M., Li H., Chen H., Zhao M., Du Y., Zhang M., Ma Y. (2022). Effect of SiO2/Al2O3 ratio on the crystallization and heavy metal immobilization of glass ceramics derived from stainless steel slag. J. Non-Crystal. Solids.

[35] Shebis Y., Vanegas A., Tish N., Fallik E., Rodov V., Poverenov E. (2022). Facile method for preparation of oligo-carboxymethyl cellulose and other oligosaccharides: Physicochemical properties and bioactivity. Food Hydrocoll..

[36] Erarpat S., Cağlak A., Bodur S., Chormey S.D., Engin Ö.G., Bakırdere S. (2019). Simultaneous determination of fluoxetine, estrone, pesticides, and endocrine disruptors in wastewater by gas chromatography–mass spectrometry (GC–MS) following switchable solvent–liquid phase microextraction (SS–LPME). Anal. Lett..

[37] Zhao C., Zhang Y., Wang C.-C., Hou M., Li A. (2019). Recent progress in instrumental techniques for architectural heritage materials. Herit. Sci..

[38] Wu J., Pan J., Wang H., Wang L., Liu W., Zhang L. (2021). Cyclone Oil Desorption Technology for the Disposal of Oil-Based Mud Cuttings. Soc. Pet. Eng. J..

[39] Wang C., Li Z., Geng X., Zhang H. (2020). Ecological Remediation of Petroleumcontaminated Soil Based on Microbial Degradation. Appl. Ecol. Environ. Res..

[40] Kuppusamy S., Maddela N.R., Megharaj M., Venkateswarlu K. (2020). Ecological impacts of total petroleum hydrocarbons. Total Petroleum Hydrocarbons.

[41] Johnston J.E., Lim E., Roh H. (2019). Impact of upstream oil extraction and environmental public health: A review of the evidence. Sci. Total Environ..

[42] Pereira L.B., Sad C.M., Castro E.V., Filgueiras P.R., Lacerda V. (2022). Environmental impacts related to drilling fluid waste and treatment methods: A critical review. Fuel.

[43] Abouelnasr D., Al Zubaidy E.A. Treatment and recovery of oil-based sludge using solvent extraction. Proceedings of the Abu Dhabi International Petroleum Exhibition and Conference, OnePetro.

[44] Wu J., Zeng L., Ma W., Zhang S., Yi X., Nie Q. (2022). Influence of cyclone oil desorption technology on the change of Oil-Based mud cuttings properties. Fuel.

[45] Fard A.K., Bukenhoudt A., Jacobs M., McKay G., Atieh M.A. (2018). Novel hybrid ceramic/carbon membrane for oil removal. J. Membr. Sci..

[46] AlHumaidan F.S., Rana M.S., Tanoli N.J., Lababidi H.M., Al-Najdi N.A. (2020). Changes in asphaltene surface topography with thermal treatment. Arab. J. Chem..

[47] Zhao M., Wang X., Liu D., Li Z., Guo S., Zhu W., Shi N., Wen F., Dong J. (2020). Insight into essential channel effect of pore structures and hydrogen bonds on the solvent extraction of oily sludge. J. Hazard. Mater..

[48] Liu K., Cheng X., Zhang C., Gao X., Zhuang J., Guo X. (2019). Evolution of pore structure of oil well cement slurry in suspension–solid transition stage. Constr. Buil. Mater..

[49] Lu T., Yuan H., Wang Y., Huang H., Chen Y. (2016). Characteristic of heavy metals in biochar derived from sewage sludge. J. Mater. Cycles Waste Manag..

[50] (2007). Hazardous Waste Identification Standards Leaching Toxicity Identification.

[51] Zhao Z., Gong X., Ding Q., Jin M., Wang Z., Lu S., Zhang L. (2021). Environmental implications from the priority pollutants screening in impoundment reservoir along the eastern route of China’s South-to-North Water Diversion Project. Sci. Total Environ..

[52] Zeng S., Ma J., Yang Y., Zhang S., Liu G.-J., Chen F. (2019). Spatial assessment of farmland soil pollution and its potential human health risks in China. Sci. Total Environ..

[53] Taha R.A., Mohamedzein Y.E.-A., Al-Rawas A.A., Al-Suleimani Y. (2010). Solidification of tank bottom sludge. Geotechn. Geol. Eng..

[54] Xiao W., Yao X., Zhang F. (2019). Recycling of oily sludge as a roadbed material utilizing phosphogypsum-based cementitious materials. Adv. Civ. Eng..

[55] Kaštanek F.E., Topka P., Soukup K., Šolcová O. (2020). Thermal treatment. The Handbook of Environmental Remediation: Classic and Modern Techniques.

